# Beyond Vessel Diameters: Non-invasive Monitoring of Flow Patterns and Immune Cell Recruitment in Murine Abdominal Aortic Disorders by Multiparametric MRI

**DOI:** 10.3389/fcvm.2021.750251

**Published:** 2021-10-25

**Authors:** Sebastian Temme, Mina Yakoub, Pascal Bouvain, Guang Yang, Jürgen Schrader, Johannes Stegbauer, Ulrich Flögel

**Affiliations:** ^1^Department of Experimental Anesthesia, Heinrich-Heine-University, Düsseldorf, Germany; ^2^Experimental Cardiovascular Imaging, Heinrich-Heine-University, Düsseldorf, Germany; ^3^Department of Nephrology, Medical Faculty, University Hospital Düsseldorf, Heinrich Heine University Düsseldorf, Düsseldorf, Germany; ^4^Department of Molecular Cardiology, Heinrich-Heine-University, Düsseldorf, Germany

**Keywords:** ^19^F MRI, monocytes/macrophages, abdominal aortic aneurysm, aortic dissection, angiotensin II

## Abstract

The pathophysiology of the initiation and progression of abdominal aortic aneurysms (AAAs) and aortic dissections (AADs) is still unclear. However, there is strong evidence that monocytes and macrophages are of crucial importance in these processes. Here, we utilized a molecular imaging approach based on background-free ^19^F MRI and employed perfluorocarbon nanoemulsions (PFCs) for *in situ* ^19^F labeling of monocytes/macrophages to monitor vascular inflammation and AAA/AAD formation in angiotensin II (angII)-treated apolipoproteinE-deficient (apoE^−/−^) mice. In parallel, we used conventional ^1^H MRI for the characterization of aortic flow patterns and morphology. AngII (1 μg/kg/min) was infused into apoE^−/−^ mice *via* osmotic minipumps for 10 days and mice were monitored by multiparametric ^1^H/^19^F MRI. PFCs were intravenously injected directly after pump implantation followed by additional applications on day 2 and 4 to allow an efficient ^19^F loading of circulating monocytes. The combination of angiographic, hemodynamic, and anatomical measurements allowed an unequivocal classification of mice in groups with developing AAAs, AADs or without any obvious aortic vessel alterations despite the exposure to angII. Maximal luminal and external diameters of the aorta were enlarged in AAAs, whereas AADs showed either a slight decrease of the luminal diameter or no alteration. ^1^H/^19^F MRI after intravenous PFC application demonstrated significantly higher ^19^F signals in aortae of mice that developed AAAs or AADs as compared to mice in which no aortic disorders were detected. High resolution ^1^H/^19^F MRI of excised aortae revealed a patchy pattern of the ^19^F signals predominantly in the adventitia of the aorta. Histological analysis confirmed the presence of macrophages in this area and flow cytometry revealed higher numbers of immune cells in aortae of mice that have developed AAA/AAD. Importantly, there was a linear correlation of the ^19^F signal with the total number of infiltrated macrophages. In conclusion, our approach enables a precise differentiation between AAA and AAD as well as visualization and quantitative assessment of inflammatory active vascular lesions, and therefore may help to unravel the complex interplay between macrophage accumulation, vascular inflammation, and the development and progression of AAAs and AADs.

## Introduction

The aorta is the largest artery in the human body that can be affected by multiple congenital or acquired diseases (1). Two important types of aortic diseases are abdominal aortic aneurysms (AAAs) and aortic dissections (AADs) which both cause >10,000 deaths in the United States each year (2). AAAs are defined as a localized enlargement of the aortic vessel wall whereas aortic dissections display a tear within the intima that causes a second lumen with flowing blood and/or intramural hematomas (1). Both, AAA and AAD can lead to aortic rupture which is associated with very high mortality (3, 4).

The exact pathophysiological mechanisms that lead to the development of AAAs or AADs have not been fully elucidated (5). For a limited number of AAAs, causative agents like infections, defects of the extracellular matrix (Marfan syndrome), or trauma have been described but a large class of AAAs seem to have nonspecific causes (6). However, in all cases, aneurysmal degeneration of the aorta involves proteolytic degradation of the extracellular matrix culminating in medial thinning and loss of structural integrity of the vessel wall. A growing number of recent studies revealed that inflammatory processes are critically involved in the pathogenesis of aortic aneurysms or dissections (7, 8). In particular monocytes, macrophages, and neutrophils but also IL17 producing Th17 cells have been shown to promote inflammatory reactions that can lead to aortic dissections or aneurysms (9–11).

Surveillance of aneurysm growth is routinely performed by ultrasound and computed tomography (CT) which provide detailed anatomical information for disease monitoring and surgical planning. However, neither modality offers insights into the pathophysiological status of the aneurysm that will determine disease progression (12). Therefore, molecular imaging approaches based on PET, MRI, or SPECT have been applied to complement the anatomical information (12, 13). ^18^F-FDG is a glucose derivative that has been explored for imaging of metabolic activity and inflammation in AAAs/AADs (14) by PET. Paramagnetic iron oxide particles (SPIOs or USPIOs) have been utilized to image the accumulation of monocytes and macrophages in aortic aneurysms using MRI (15). Further targets that have been investigated in AAAs/AADs by PET are the somatostatin receptor with ^68^Ga-DOTATE (16), the translocator protein by ^11^C-PK11195 (17) or the choline receptor with ^18^F-FMCH (18).

A more recently established technique to visualize inflammatory processes is based on the MR-active nucleus fluorine 19 (^19^F) (19). ^19^F has the second highest sensitivity among all MR nuclei, a natural abundance of 100 % and is nearly absent from biological tissue (20). Therefore, the accumulation of ^19^F atoms in the body can be detected with high specificity and sensitivity. Chemical compounds with high ^19^F content are perfluorocarbons which are chemically and biologically inert but must be emulsified with lipids to generate biocompatible perfluorocarbon nanoemulsions (PFCs) (21). Intravenous application of PFCs leads to phagocytic uptake predominantly by monocytes and macrophages which accumulate at the inflammatory hot spot and can be detected by combined ^1^H/^19^F MRI (22–24). Of note, ^19^F MRI does not only allow for detection of the anatomical localization of infiltrated cells, the ^19^F signal can also be used to quantify cell numbers since there is a linear relationship between the amount of ^19^F atoms and the ^19^F signal.

Due to the importance of inflammation in the development and progression of AADs/AAAs, the present study aimed to utilize PFCs in combination with ^1^H/^19^F MRI to visualize inflammatory processes and the accumulation of monocytes and macrophages in the aortic vascular wall of angiotensin II (angII) treated apoE-deficient (apoE^−/−^) mice, a well-established mouse model known to result in a portion of animals in AAD and AAA (25, 26).

## Materials and Methods

### Animal Ethics

All experiments were performed in accordance with the German law for animal protection and were approved by the local ethic committees (file reference G301/18). ApoE^−/−^ mice (Taconic, Denmark) were bred and housed at the central animal facility (ZETT) at the Heinrich-Heine-University (Düsseldorf, Germany) and maintained on a 12:12 h day:night cycle with constant access to food and water. In anesthetized mice (ketamine 100 mg/kg and xylazine 10 mg/kg, intraperitoneal), osmotic micropumps (Alzet, Model 1002) were implanted subcutaneously to infuse angII (1 μg/kg/min) chronically to apoE^−/−^ for 10 days. To conduct ^1^H/^19^F MRI, PFCs (3 mmol/kg/BW) were injected intravenously *via* the tail vein in anesthetized (1.5% isoflurane) mice.

### Preparation and Characterization of Perfluorocarbon Nanoemulsions

Perfluorocarbon nanoemulsions (PFCs) were essentially prepared as described previously (22, 24). In brief, lipids E80S (35 mM) (Lipoid GmbH) were dissolved in phosphate glycerol buffer, perfluoro-15-crown-5 ether was added, and the mixture was processed by high-shear mixing (Ultraturrax) to form a pre-emulsion. The pre-emulsion was then subjected to microfluidization (M110P, Microfluidics) for five cycles at 1,000 bar. PFCs were transferred to glass vials and sterilized by autoclaving at 121°C, 1 bar for 20 min, and subsequently analyzed by dynamic light scattering for quality control (see below).

#### Dynamic Light Scattering

The mean intensity-weighted hydrodynamic diameter was determined on a Nanotrac Wave II (Microtrac MRB). Prior to measurements, the nanoemulsions were diluted 1:100 (v/v) with MilliQ water. Data acquisition was performed at 25°C and at a scattering angle of 173°. From this the following parameters were determined as described previously (27): particle size as averaged hydrodynamic diameter (d_z_); width of the particle size distribution as polydispersity index (PDI) and zeta (ζ) potential in mV. Size and PDI were determined in five measurements each consisting of five sub-runs, measurements of the ζ potential were performed with the same sample thereafter.

### Magnetic Resonance Imaging

Experiments were performed at a vertical 9.4 T Bruker AVANCE^III^ Wide Bore NMR spectrometer (Bruker) operating at frequencies of 400.21 MHz for ^1^H and 376.54 MHz for ^19^F measurements using microimaging units as described previously (22). Mice were anesthetized with 1.5% isoflurane and were kept at 37°C during the measurements. For gated MRI acquisitions, the front-paws and the left hind-paw were attached to ECG electrodes (Klear-Trace) and respiration was monitored by means of a pneumatic pillow positioned at the animal's back. Vital functions were acquired by a M1025 system (SA Instruments) and used to synchronize data acquisition with cardiac and respiratory motion. Data were acquired using a 25-mm quadrature resonator tuneable to ^1^H and ^19^F. After acquisition of the morphological ^1^H images, the resonator was tuned to ^19^F and anatomically matching ^19^F images were recorded. The reference power and the receiver gain were kept constant between the measurements to ensure comparability of the ^19^F scans.

#### *In vivo* ^1^H MRI

To visualize the ***anatomy of the region of interest***, ^1^H MR reference images from the abdomen were acquired using a rapid acquisition and relaxation enhancement sequence [RARE; field of view (FOV) = 2.56 × 2.56 cm^2^, matrix = 256 × 256, 0.1 × 0.1 mm^2^ in plane resolution, 1 mm slice thickness (ST); repetition time (TR) = 2,500 ms; RARE factor = 16, 6 averages (NA), acquisition time (TAcq) = ~5 min]. ^1^H MR ***time-of-flight angiography*** to visualize dilatation or narrowing of the aorta *via* its blood flow pattern was carried out by a ^1^H fast low angle shot (FLASH) 2D flow compensated sequence; FOV = 2.56 × 2.56 cm^2^, matrix = 256 × 256, 0.1 × 0.1 mm^2^ in plane resolution, ST = 0.5 mm; 0.25 mm overlap, 100 slices; TR = 10 ms; NA = 6; TAcq = 4 min). ***Aortic bright blood cine movies*** were acquired in sagittal orientation adapted to the anatomical course of the vessel using an ECG- and respiratory-gated segmented fast gradient echo cine sequence with steady state precession (FISP). A flip angle (FA) of 15°, echo time (TE) of 1.23 ms, and a TR of about 6–8 ms (depending on the heart rate) were used to acquire 16 frames per heart cycle from a FOV of 30 × 22 mm^2^, matrix = 256 × 192, ST = 1 mm, NA = 3, TAcq per slice for one cine loop ~2.5 min. From the same FOV, corresponding ***black blood cine movies*** were recorded utilizing an ECG- and respiratory-gated FLASH sequence with black blood preparation (blood inversion time ~100 ms and TR ~8 ms depending on the heart rate, TE = 2.28 ms, matrix = 256 × 192, ST = 1 mm, 16 frames, NA = 4, TAcq ~5 min. ***Aortic flow profiles*** were obtained by acquisition of velocity maps at the suprarenal level. Measurements were performed using an ECG- and respiration-triggered slice-selective FLASH sequence with a four-point Hadamard scheme for flow velocity encoding (28). Twelve frames per heart cycle were acquired using the following parameters: TE/TR = 1.75/7.50 ms, FA = 30°, FOV = 30 × 30 mm^2^, ST = 1 mm; matrix = 256 × 256; NA = 4 resulting in a TAcq of ~5 min.

#### *In vivo* ^1^H/^19^F MRI

Anatomically matching ^19^F images were recorded from the same FOV with a ^19^F RARE sequence (matrix = 64 × 64, 0.4 × 0.4 mm^2^ in plane resolution, ST = 1 mm, TR = 4,000 ms, RARE factor = 32, NA = 25, and TAcq = 34 min).

#### High Resolution ^1^H/^19^F Post-mortem

After the last *in vivo* MRI session, the aorta was carefully excised, cleaned from fat and connective tissue, fixed in paraformaldehyde, and finally embedded in agarose to avoid any motion throughout the ^1^H/^19^F MRI measurements. Three-dimensional turbo RARE ^1^H/^19^F sequences were used to image the excised and embedded vessels. ^1^H: FOV = 1.00 × 1.00 cm^2^, matrix = 128 × 128, 0.078 × 0.078 mm^2^ in plane resolution, ST = 0.156 mm; TR = 1,500 ms; NA = 8, and TAcq = 5 h). ^19^F:FOV = 1.00 × 1.00 cm^2^, matrix = 64 × 64, 0.156 × 0.156 mm^2^ in plane resolution, ST = 0.31 mm; TR = 1,500 ms; NA = 180, and TAcq = 14.5 h).

#### Data Analysis

To quantify the luminal or the external diameter of the aorta, the cross section either of the flowing blood (luminal diameter) or of the aortic wall based on the anatomical scans was determined in Fiji (29) using the Bruker Plugin (30). Since the aortic wall is only 100 μm in diameter, which is hardly detectable by conventional ^1^H MRI, we determined the extension of the external diameter. For quantification of flow velocities, aortic demarcations were manually drawn with the ParaVision ROI tool to calculate mean and maximal velocities of the ROI. For 3D surface visualization, aortic flow profiles were extracted from the dataset by an in-house developed software module based on LabVIEW and plotted with OriginPro (Originlab Corporation).

The ^19^F MR data was quantified using Fiji (29) and appropriate Bruker plugins (30). For quantification of the ^19^F MRI data, ROIs were drawn around the ^19^F signal to determine both the mean and total ^19^F intensity. Background ROIs were placed outside the animals. The SNR was calculated by: ^19^F SNR = (^19^F signal intensity–mean background signal)/standard deviation of the background signal (noise). Three-dimensional reconstruction of the high-resolution datasets was done with Amira (Mercury Computer Systems). Here, the outer area of the vessel wall and the lumen were manually segmented and the ^19^F signal was superimposed to the transparent volume rendering of the vessel anatomy.

### Flow Cytometry and Histology

#### Flow Cytometry

Animals were anesthetized using a mixture of ketamine (100 mg/kg, i.p.; Ketaset, Zoetis) and xylazine (10 mg/kg, i.p.). Aortae, perfused with ice-cold PBS, were carefully dissected, cut into small pieces, and incubated in a collagenase-containing digestion solution (600 U/ml collagenase type II, 60 U/ml DNase I, in HBSS) for 60 min at 37°C. The suspension was passed through a 70 μm filter and cells were pelleted by centrifugation and washed twice with MACS buffer. Cells were stained with mAbs against CD45, CD11b, Ly6G, Ly6C, and F4/80 to identify total immune cells, monocytes (CD45+, CD11b+, Ly6G–, and Ly6C+), neutrophils (CD45+, CD11b+, and Ly6G+) and macrophages (CD45+, CD11b+, Ly6G–, and F4/80+).

#### Movat Staining

Movat staining was performed as described previously (31). In brief, transverse sections of formalin-fixed aortae were cut for histological staining. Microscopical slides were fixed in Bouin's solution for 10 min at 50°C, immersed in 5% sodium thiosulfate for 5 min, 1% Alcian blue for 15 min, and then alkaline alcohol for 10 min at 60°C. Movat Weigert's solution was prepared out of 2% alcohol hematoxylin, ferric chloride stock solution, and iodine stock solution in a ratio of 3:2:1. Tissues were stained in Weigert's solution for 20 min and subsequently in crocein scarlet acid/fuchsin working solution (3:1) for 2 min. Thereafter, the slices were placed in 5% phosphotungstic acid for 5 min and then transferred immediately in 1% acetic acid for 5 min. Dehydration was carried out in 95 and 100% ethanol, respectively. Slices were immersed in alcohol saffron for 8 min, twice in 100% ethanol for 1 min, and then twice in xylol for 5 min. Finally, tissues were mounted and covered by coverslips. Chemicals were purchased from Sigma, Chempur, Microm, and Carl-Roth.

#### F4/80 Staining

Histological slices were incubated in proteinase K (S3020, Dako) for 2.5–3 min at room temperature. Afterward, slides were loaded with 3% H_2_O_2_ for 10 min and thereafter with horse serum (Vector, MP-7401 Burlingame, CA, USA) for 20 min. Without rinsing, slides were incubated in monoclonal rat-anti mouse F4/80 antibody (1:100, MCA497RT, Bio-Rad) overnight at 4°C. After extensive washing with buffer (Dako, S0809), slides were incubated with ImmPRESS anti-rabbit IgG HRP (No dilution, Vector, MP-7401) for 30 min at room temperature. The staining was visualized using DAB/HRP stain (DM827, Agilent). After washing with tap water for 10 min, slides were dried and mounted.

### Statistics

Statistical analyzed were performed with “R” (32) or Prism6 (GraphPad Software). Data were first analyzed for normality using Shapiro–Wilk and then further analyzed using a student's *t*-test, Welch's-test or Mann–Whitney-test if the data points were not normally distributed.

## Results

### Classification of Developing Aortic Disorders After angII Exposure

ApoE-deficient mice (apoE^−/−^) were treated with angII for 10 days and subsequently subjected to anatomical and angiographic ^1^H MRI to monitor aortic vessel alterations. The pulse sequence used for anatomical imaging (RARE) results in a signal void of flowing blood, allowing to resolve more clearly pathological structures in the vessels (e.g., thrombi), while in time-of-flight images just flowing blood is visible.

**Figure 1:**
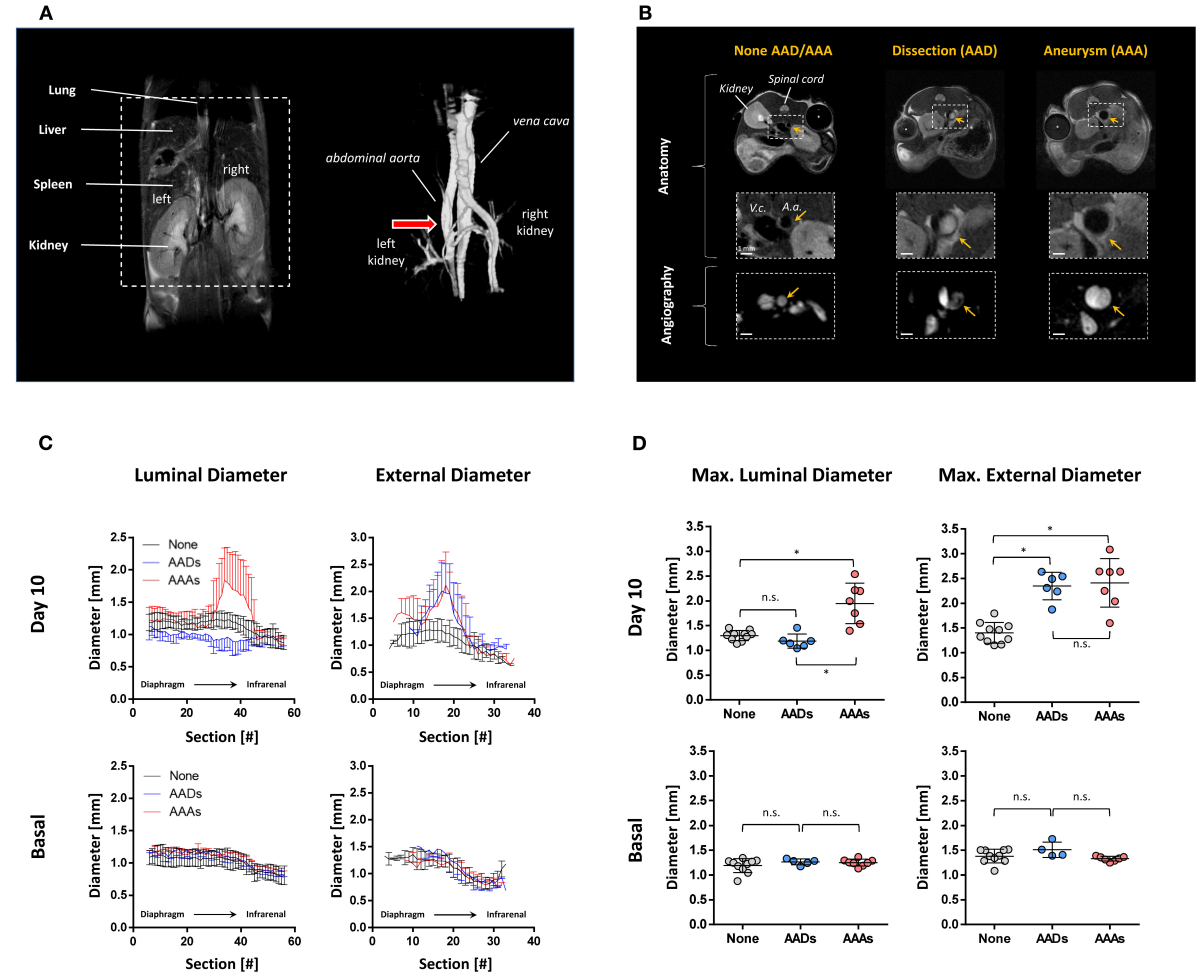
^1^H MRI of angII-induced aortic disorders. **(A)** Coronal ^1^H MRI of the abdominal area (left) and a 3D reconstruction of the corresponding ^1^H MR angiography (right). The dashed lines represent the area inspected for the analysis of AAA and AAD. The red arrow highlights the site where AAAs/AADs predominantly develop. **(B)** Upper panel: Axial ^1^H MRI cross sections of mice treated with angiotensin II for 10 days. Middle: Magnification of the aortic area indicated by the dashed lines. Lower: Corresponding MR angiographic slices. Yellow arrows indicate the site of the aorta. None, mouse without developing AAA/AAD phenotype. **(C)** Quantification of the luminal and external anteroposterior diameters of the aorta before implantation of angII minipumps (basal) and on day 10 of angII treatment. The number of sections indicate the position of the scans from cranial (diaphragm. i.e., #1) to caudal (~bottom of kidneys, i.e., #40). **(D)** Maximal diameter of the aortic lumen or the external vessel wall at baseline and day 10 of angII treatment, respectively. None, mice with normal anatomy and angiography of the aorta; AAD, aortic dissection; AAA, aortic aneurysm. Data are statistically significant with ^*^p ≤ 0.05.

Figure 1A (left) shows a representative coronal cross section of the murine abdomen and the dashed lines represent the area that was analyzed by MR angiography (MRA). Three-dimensional reconstruction of such an angiogram is displayed on the right with the arrow pointing to the suprarenal area of the aorta prone to the development of aortic disorders upon angII exposure. The combined acquisition of morphological and angiographic ^1^H MRI scans allowed an easy differentiation of mice with AADs and AAAs (Figure 1B) from those without aortic disorder despite angII exposure. AAAs are characterized by an eccentric increase in vessel wall diameter (Figure 1B, right, top/middle) with detectable time-of-flight signal (i.e., blood flow) over the entire lumen (Figure 1B right, bottom), On the other hand, AADs are frequently associated with large intramural thrombi which can be recognized in the anatomical images as medium gray structure within the aortic lumen (Figure 1B, middle, top/middle) leading to areas of diminished or completely lacking flow in angiograms (Figure 1B, middle, bottom). In the example given, parts of the thrombus area exhibit some time-of-flight signal suggesting persisting partial perfusion of this region.

Quantification of the vessel diameter in aortic time-of-flight cross sections from the diaphragm to ~2.5 cm below the left renal artery showed an increase in the luminal diameter for AAAs in the suprarenal region (sections 10–30), whereas the diameter for AADs was slightly reduced in this range compared to mice without AAD/AAA (Figure 1C, right). However, the external diameter as derived from RARE images was enlarged for both AAAs and AADs in the same area (Figure 1C, left). Thus, the comparison of the maximal luminal and external diameters allowed unequivocal discrimination between AAAs and AADs (Figure 1D). Compared to mice which did not develop any visible aortic vessel alterations (luminal: 1.3 ± 0.1 mm; external: 1.4 ± 0.2 mm) both luminal and external diameter was increased in AAAs (luminal: 1.9 ± 0.4 mm; external: 2.4 ± 0.5 mm) whereas only the external diameter was enlarged in AADs (luminal: 1.2 ± 0.1 mm; external: 2.3 ± 0.3 mm) (Figure 1D). Of note, the anatomy of the abdominal aorta before implantation of osmotic minipumps was similar for all groups in both time-of-flight and RARE images (Figures 1C,D, lower panel).

### Differentiation of Hemodynamic Alterations Associated With AAAs and AADs

**Figure 2:**
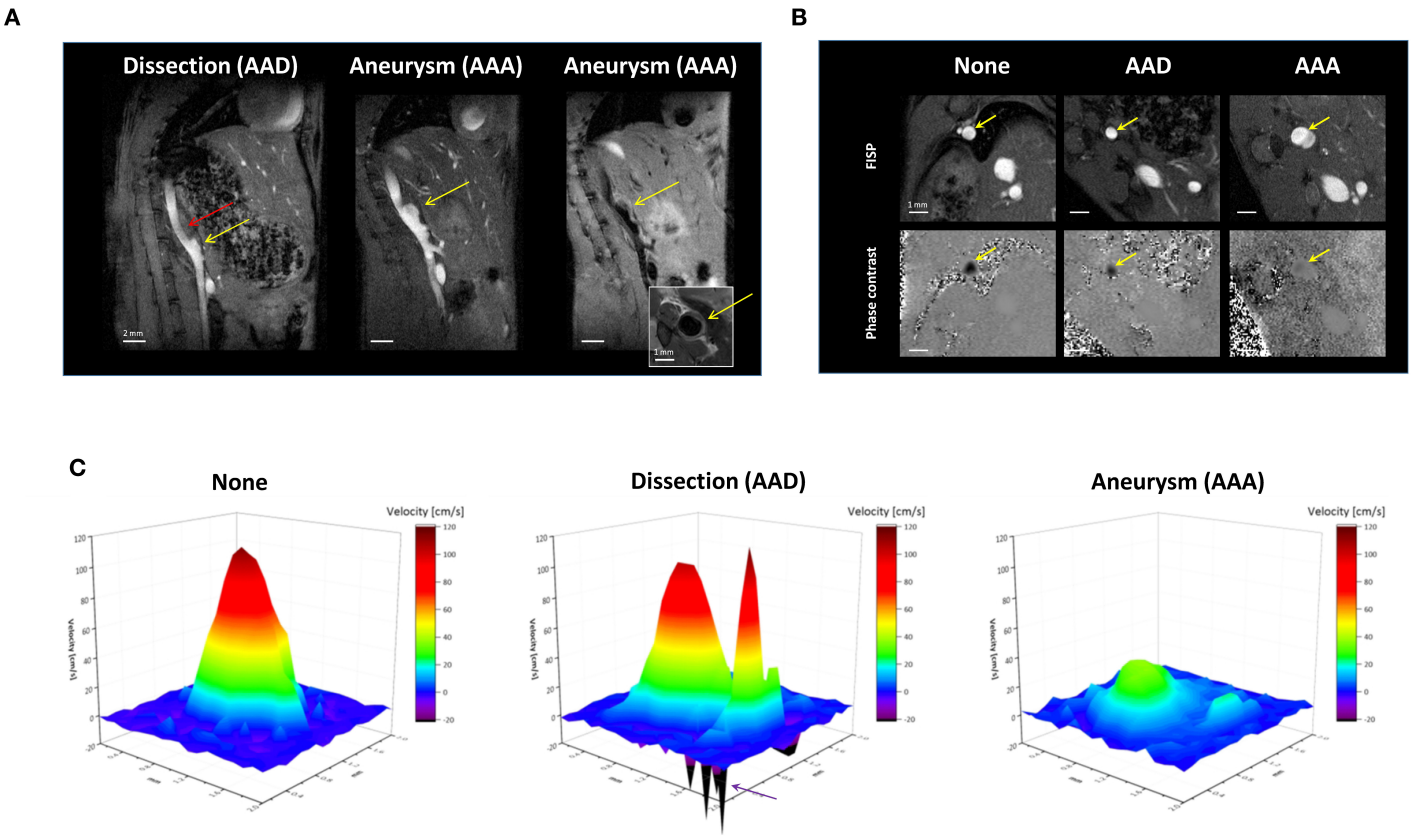
Hemodynamic consequences of angII-induced vessel alterations. **(A)** End-diastolic sagittal images from ^1^H cine bright blood movies in AAD (left) and AAA (middle). Right: Corresponding ^1^H black blood images acquired in the same orientation as in the adjacent bright blood image as well as in perpendicular orientation (insert). **(B)** Upper panel: Cross-sectional bright blood images of mice without AAA/AAD (None) and with AAD (middle) or AAA (right). Lower panel: Anatomical corresponding phase contrast MR images acquired in end-systole. **(C)** 3D surface plot of the flow velocity profiles shown in **(B)**. Normal aorta (control, left); AAD (middle) and AAA (right). The purple arrow marks the sites of reverse flow.

To further characterize the impact of the different luminal diameters—as one of the hallmarks in AAAs/AADs—on aortic hemodynamics at the lesion site, we monitored blood flow dynamics in the aorta. For this purpose, we acquired ECG- and respiratory-gated cine movies with bright or black blood preparation and flow velocity encoding, respectively, over the entire cardiac cycle. Figure 2A shows sagittal bright blood ^1^H MR images of the abdomen adapted to the anatomical course of the aorta from mice with AAD (left) or AAA (middle+right). As can be clearly recognized, in AAD an extension of the lumen developed only over a small part of the vessel (yellow arrow). However, above this area a large thrombus evolved (red arrow) occupying almost half of the vessel diameter. In contrast, in AAA a massive bulging of the lumen is visible (yellow arrow) in both bright and black blood images of the same orientation (Figure 2A, middle+right). Of note, the corresponding axial black blood view gave no evidence for the presence of a thrombus in AAA (Figure 2A, right, insert) but, interestingly, the bright blood image at the same position shows a distinct demarcation of the greyscale gradation in the middle of the vessel (Figure 2B, right, top) suggestive of different blood flow velocities or directions.

Cine movies confirmed that these anatomical differences have a massive impact on the local flow dynamics in the aorta (Supplementary Movie 1). In bright blood cine movies, the intensity of the black jet artifacts goes along with increased blood flow velocities, thus indicating faster flow in AADs compared to AAAs. This is clearly corroborated by cine velocity maps acquired perpendicular to the longitudinal image plane in Figure 2A. Figure 2B bottom displays representative phase contrast images from these measurements acquired at peak flow—in this part of the aorta usually ~50 ms after detection of the QRS complex (≈end-systole); anatomical matching bright blood images are illustrated in Figure 2B top for better orientation. As expected, 3D surface plots of these data (Figure 2C) showed a normal laminar and bell-shaped flow profile with a peak velocity around 100 cm/s for mice without aortic disorder (left). In contrast, both AAD and AAA were accompanied by irregular flow profiles with splitted maxima. As already indicated by the bright blood cine movies, in AAD peak flow velocities were almost unaltered but the splitted flow components were associated with fragmented turbulent patterns and isolated spikes of negative flow peaks around the dissection (Figure 2C, middle, arrow). In contrast, dilated areas in AAAs exhibited a strongly decreased peak flow velocity down to 20 cm/s (Figure 2C, right).

### Monitoring Vascular Inflammation in Aortic Disorders by Combined ^1^H/^19^F MRI

**Figure 3:**
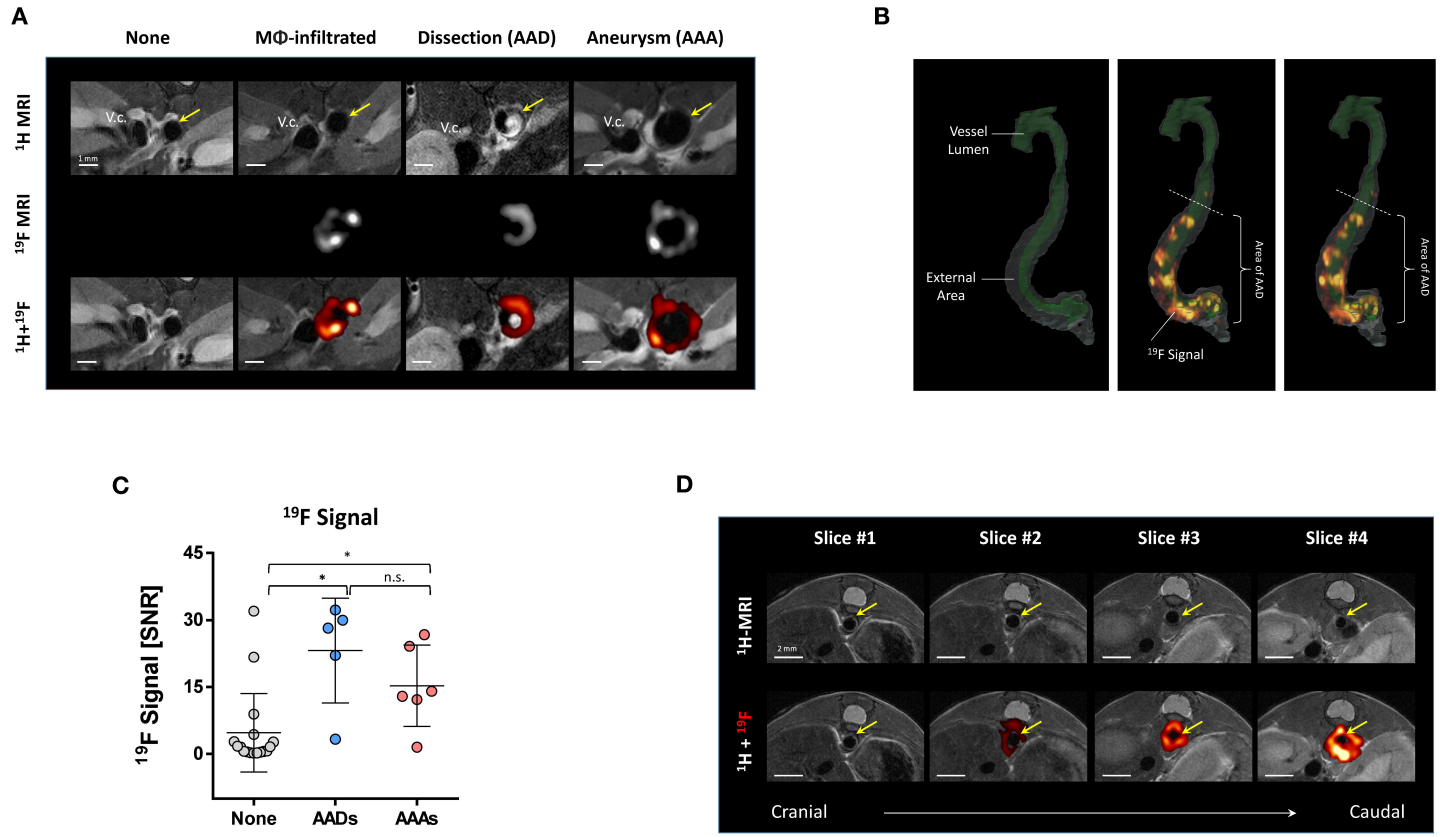
^1^H/^19^F MR inflammation imaging in AAAs and AADs. **(A)** For imaging of inflammation, perfluorocarbon nanoemulsions (PFCs) were injected intravenously for *in situ* labeling of monocytes/macrophages followed by ^1^H/^19^F MRI. Displayed are axial ^1^H scans of the abdominal area (upper panel), the anatomically matching ^19^F images (middle) as well as a merging of ^1^H and the aortic ^19^F signal (hot iron scale; lower panel). None, healthy animal without AAA/AAD and no vascular ^19^F signal; MΦ-infiltrated, no presence of AAD/AAA despite angII treatment but ^19^F signal around the vascular wall; AAD/AAA, abdominal aortic dissections and aneurysms. Arrows indicate the site of the abdominal aorta. **(B)** 3D reconstruction of a segmented high-resolution *ex vivo* ^1^H/^19^F MRI scan from an excised and paraformaldehyde fixed aorta with aortic dissection. Gray, external area of the vessel; green, lumen of the vessel; “hot-iron”, ^19^F signal. The white dashed line indicates the beginning of the dissection. **(C)** Quantification of the ^19^F signal (mean ^19^F signal-to-noise ratio) around the vascular wall. **(D)** ^1^H/^19^F MRI of an area that is above the location of an aortic dissection. Upper, ^1^H MRI; lower, merging of ^1^H and ^19^F MRI data. ^19^F signals of the liver and the spleen were faded out for the sake of clarity. The sequence of the images from left to right is from cranial to caudal. The arrows mark the abdominal aorta (A.a) and the beginning of the aortic dissection can be recognized in the right image as dark structure with an intense ^19^F signal. Data are statistically significant with ^*^p ≤ 0.05.

To assess vascular inflammation during the early course of angII treatment, we injected perfluorocarbon nanoemulsions (PFCs) intravenously 2, 48 h as well as 96 h after minipump implantation for ^19^F loading of circulating immune cells. ^1^H/^19^F MRI was not conducted until 48–72 h later to enable an adequate infiltration and accumulation of labeled phagocytic cells (see Supplementary Figure 1 for an overview of the experimental setup). Typical background-free ^19^F MR images are displayed in Figure 3A middle together with their corresponding anatomical ^1^H MR reference images Figure 3A top. Merging of both images (with ^19^F data encoded in red) revealed that mice with developing AAD or AAA exhibited strong ^19^F signals around the aortic vascular wall (Figure 3A, right). Of note, the resulting ^19^F patterns in the vessel wall were found to be quite diverse without any obvious correlation with the aortic disorder type. To gain more precise information about the spatial localization of the ^19^F signal, we used high resolution ^1^H/^19^F MRI of PFA-fixed aortae. Different views on the 3D reconstruction of the ^1^H/^19^F MRI datasets are shown in Figure 3B providing a general survey of the ^19^F signal distribution along the entire abdominal aorta. These highly resolved images clearly confirmed a peripheral location of the ^19^F signal outside the vessel lumen (green) and in the external area of the vessel wall (gray; see also Supplementary Movie 2). However, the signal distribution appears here much more patchy as could be derived from the *in vivo* data (Figure 3A).

Whereas, most of the animals without AAA/AAD showed no ^19^F signal in the aortic wall, some mice had interestingly quite strong ^19^F signals in the aortic wall indicating acute inflammation without any obvious alterations in the anatomical ^1^H MR image of the vascular wall (Figure 3A, 2nd column). Nevertheless, quantification of all data demonstrated that the ^19^F signal was significantly higher in the aortic vessel wall in AAAs and AADs compared to unaffected aortas (Figure 3C). Here, we noticed pronounced ^19^F signals already 12-24 h after intravenous injection, but, interestingly, thereafter only minor alterations in the ^19^F signal were observed (data not shown), suggesting a constant pool of ^19^F-loaded macrophages in the aortic wall. The spatial distribution and the intensity of the ^19^F signal were similar in AAAs and AADs with most of the ^19^F signal located in the adventitia of the vessel wall whereas the intraluminal thrombus of AADs seemed to lack any ^19^F signal.

Of note, in some mice we also observed ^19^F signals in anatomically inconspicuous regions outside the area of AAAs/AADs. In Figure 3D, four slices in caudal direction ending at the upper end of an aortic dissection (slice #4) are shown. In slice #4 at the beginning of the AAA, one can recognize the thrombus as part of the AAD together with strong ^19^F signals. However, the slices #1–#3 above the dissection do not show any obvious changes in the anatomical ^1^H MRI images, but nevertheless clear ^19^F signals around the aortic wall are visible.

### The Vascular ^19^F MRI Signal Correlates With Localization and Number of Macrophages

**Figure 4:**
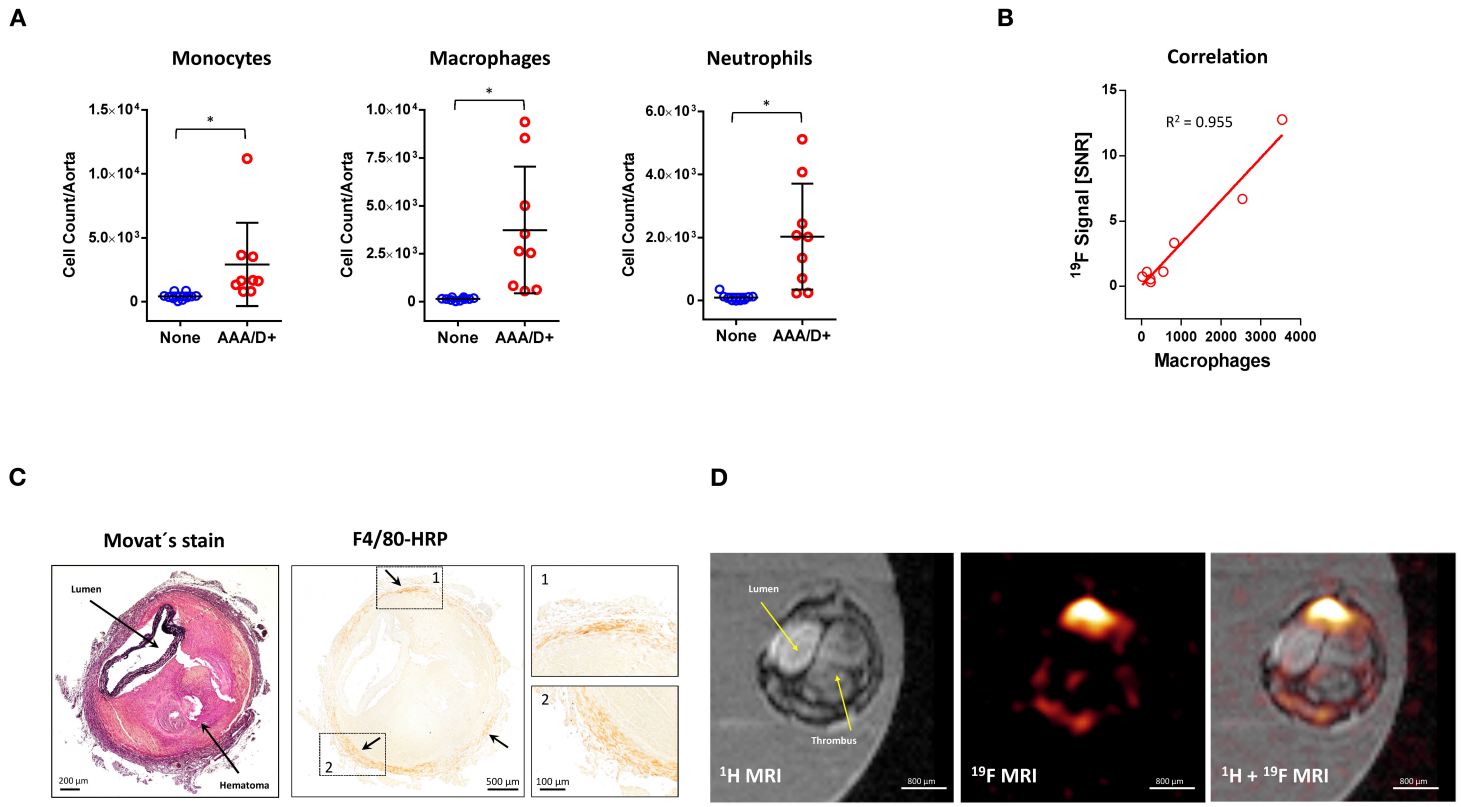
Flow cytometric and histological validation of *in vivo* MRI data. **(A)** Flow cytometry of myeloid immune cells isolated on day 10 of angII treatment from aortae without AAA/AAD or vascular inflammation (None) and aortae with AAA/AAD (AAA/D+). **(B)** Correlation of the vascular ^19^F signal with the total number of aortic macrophages determined by flow cytometry. **(C)** Histological sections were stained with Movat's (left) or incubated with antibodies against F4/80 followed by horseradish peroxidase (HRP) reaction (right). Arrows show the localization of the brown HRP-positive signals. The dashed lines indicate the magnified areas on the right **(D)** High resolution *ex vivo* ^1^H/^19^F MRI measurements of an excised and paraformaldehyde fixed aorta with AAD that was embedded in agarose. Data are statistically significant with ^*^p ≤ 0.05.

To obtain further information about the cellular and structural origin of the vascular ^19^F signal, we performed flow cytometry as well as histological analysis of aortae with or without aneurysms or dissection. Flow cytometric analysis of the number of immune cells isolated from vessels with AAA/AAD and without aortic disorder revealed a significant increase in the numbers of monocytes, macrophages and neutrophils in those aortae that exhibit an AAA/AAD phenotype (Figure 4A, red). Aortae without AAA/AAD and without strong vascular ^19^F signals showed only low numbers of immune cells (Figure 4A, blue). Importantly, linear regression of the ^19^F data with the number of macrophages obtained by flow cytometry revealed an excellent correlation (*R*^2^ = 0.955) between the ^19^F signal and the macrophages count within the vessel wall (Figure 4B).

Movat's staining of histological sections was utilized to visualize the soft tissue structures of the AADs and confirmed large intramural hematoma (light red = fibrin) located between the media and adventitia (Figure 4C, left) as also observed *in vivo* by ^1^H MRI (Figures 1–3). Note that elastin fibers of the media (black) are still largely intact. Furthermore, F4/80 staining (dark brown signal) of tissue sections was used to display macrophages. Here, the signal was multifocal and localized predominantly within and around the adventitia (Figure 4C, right).

To reconcile these histological findings with the MRI data, we analyzed high resolution ^1^H/^19^F MRI data of this PFA-fixed aorta, that was acquired before histology. In the ^1^H MR image (Figure 4D, left), vessel lumen, the large intramural hematoma, and the external part of the vessel wall can be clearly recognized. The corresponding ^19^F data (Figure 4D, middle) revealed a patchy circular ^19^F pattern which is predominantly located around the adventitia (Figure 4D, right) and is nearly absent from the hematoma. Comparison of the histological sections with the axial ^1^H/^19^F images strongly indicates that the ^19^F signals match the localization of F4/80^+^ macrophages.

## Discussion

In the present study, we investigated the feasibility to visualize vascular inflammation during development of abdominal aortic disorders by non-invasive, multiparametric MRI. Using anatomical and angiographic ^1^H MRI including aortic flow patterns, we could easily differentiate between the formation of AAAs and AADs over time. ^19^F MRI allowed the *in vivo* monitoring of vascular inflammation *via* detection of strong ^19^F signals in the aortic wall after *in situ* labeling of monocytes/macrophages by intravenous PFCs application. *Post mortem* high resolution ^1^H/^19^F MRI, histology, and flow cytometry confirmed that localization and intensity of the ^19^F signal matched the location and number of monocytes/macrophages within the aortic wall. The regular and early detection of ^19^F-loaded monocytes/macrophages in mice with AAD/AAA corroborated that vessel inflammation plays a crucial role in driving aortic disease development. However, the occasional occurrence of ^19^F signal in mice without AAD/AAA or in regions above or below the lesion suggests that in some cases aortic inflammation might occur prior to or independent from vascular pathology and that additional factors are required for manifestation of an apparent aortic disorder.

Noninvasive imaging of inflammatory processes by combined ^1^H/^19^F has gained increasing interest over the past years (19, 33). The ^19^F nucleus has the second highest sensitivity among all NMR active nuclei, has a natural abundance of 100% and is nearly absent from normal tissue (20). Therefore, the local deposition of ^19^F atoms can be visualized by ^1^H/^19^F MRI with high sensitivity and specificity (19). Here, we used lipid-stabilized perfluorocarbon nanoemulsions (PFCs) that are known to be avidly taken up by monocytes and macrophages (23, 33). In angII-treated apoE^−/−^ mice, a well-established animal model to investigate the pathogenesis of AAA and AAD (25), we observed that the ^19^F signal is predominantly located in the external part of the aortic wall and matched with the localization of F4/80^+^ macrophages. Importantly, there was a linear correlation between the total number of macrophages and the ^19^F signal suggesting that signal strength is an indicator for the number of infiltrated phagocytes. This in line with previous studies which have shown that in cardiac, pulmonary, or intestinal inflammation the ^19^F signal reflects the number of infiltrated monocytes and macrophages (22, 34–37). Specifically, in a mouse model of inflammatory bowel disease, Kadayakkara et al. (35) revealed a strong correlation between F4/80^+^ macrophages and the localization of PFCs, while deletion of monocytes/macrophages strongly reduced the ^19^F signal in the gut. Similar observations were also made in murine models of myocardial infarction or myocarditis where the vast majority of the ^19^F signal within the heart was associated with cardiac monocytes/macrophages (36, 37). However, it should be noted that other phagocytic cells like neutrophil granulocytes (24, 34, 38), B-cells (22) can also internalize PFCs and that under certain circumstances, a significant amount of the ^19^F signal can be derived from neutrophil granulocytes (24, 38) or even progenitor cells in the heart (39). Furthermore, it must be considered that parts of the ^19^F label within macrophages can be derived from neutrophils which are internalized by macrophages by efferocytosis during the resolution process. However, despite these previous reports on inflammatory processes in rather big organs (heart, lung, and gut) the present study is the first, which could visualize vascular inflammation in the ~0.1 mm thin aortic wall *in vivo* and corroborate that infiltrating immune cells play a key role during development of abdominal aortic diseases in mice.

Interestingly, the heterogenous appearance and anatomy of aortic disorders seen in anatomic and angiographic MRI sequences are also reflected by the spatial distribution of the ^19^F signal. In both AAA/AAD, the ^19^F signal was patchy distributed across the aortic wall suggesting that vascular inflammation in AAA or AAD manifests in local hot-spots rather than being a homogeneous process. This observation is again in agreement with previous studies that have observed a heterogenous distribution of both the ^19^F signal and macrophage distribution in the inflamed gut (35), the infarcted heart (22, 36) and experimental viral myocarditis (37). Within the aortic wall, macrophages execute multiple functions since they contribute to the degradation of the extracellular matrix (40, 41), to the modulation of the inflammatory response (10) and also to the regulation of tissue healing and repair (7, 8). Thus, it is conceivable that these local ^19^F hot spots could indicate either an area of biologically active macrophages with tissue degrading profile that foster aortic rupture but also a tissue stabilizing anti-inflammatory milieu. Clearly, for more insight into the exact underlying pathomechanisms, further investigations are needed.

In recent years, substantial progress has been achieved in targeting of PFC nanoparticles for the identification of specific immune cell subsets with different properties. Thus, specific coupling to PFC to distinct immune cell subsets will path the way for a more specific monitoring of infiltration kinetics and contribution of these individual subpopulations to disease progression or healing. Specific visualization of macrophage subtypes like pro-inflammatory M1 or pro-healing M2 macrophages could add important information regarding the polarization of the inflammatory response which has a significant impact on disease progression and severity (42, 43). Of note, distinct labeling and detection of M2 macrophages by positron emission tomography (PET) or near infrared fluorescence imaging (NIRF) has been performed by targeting the mannose receptor CD206 (44–47). Since, we and others have recently provided evidence that it is possible to retarget PFCs from phagocytic cells and to enable a specific visualization of early thrombi (48), activated platelets (49), or specific cell types (50), a similar approach could also be pursued by ^19^F MRI and would be an interesting option for the future to gain more insight into the pathophysiology of AAAs/AADs.

Our approach can be further expanded by longitudinal multiplex ^19^F MRI to visualize multiple cell types or targets (51–56) and combined with parametric and functional MRI. T1 as well as T2 mapping, late gadolinium enhancement, or chemical exchange saturation transfer MRI could be carried out in parallel to ^19^F MRI to obtain a comprehensive overview of the evoked immune response and development/progression of tissue damage over time (57, 58). Moreover, the co-registration of local flow patterns will provide additional information about vulnerable regions in the aorta prone to rupture.

In summary, multimodal ^1^H/^19^F-based MRI holds the potential to connect preclinical as well as clinical studies to further unravel the complex mechanisms leading to AAAs/AADs, to discriminate inflammatory bioactive and dormant AAAs/AADs, and to monitor the efficacy of novel therapeutic approaches. Finally, the additional loading of targeted PFCs with drugs will allow the selective delivery of pharmaceuticals to abdominal aortic foci of inflammation as a therapeutic option.

## Data Availability Statement

The original contributions presented in the study are included in the article/Supplementary Material, further inquiries can be directed to the corresponding author/s.

## Ethics Statement

The animal study was reviewed and approved by Landesamt für Natur, Umwelt, und Verbraucherschutz (LANUV), Nordrhein-Westfalen, Germany.

## Author Contributions

ST, MY, PB, GY, JSt, and UF performed experiments, analyzed, and interpreted data. ST, MY, JSt, and UF prepared the manuscript. JSc critically revised the manuscript, provided expert advice, and interpreted data. All authors contributed to the article and approved the submitted version.

## Funding

This work was supported by the Deutsche Forschungsgemeinschaft (TE1209/1–1+2 to ST; IRTG1902, and STE2042–2/1) to JSt; SFB 1116 to JSc and UF; TRR 259 to UF; FL303/6–1+2 to UF; INST 208/764-1 FUGG to UF) and the European Commission (MSCA-ITN-2019, NOVA-MRI to JSc and UF; MSCA-RISE-2019, PRISAR2 to UF).

## Conflict of Interest

The authors declare that the research was conducted in the absence of any commercial or financial relationships that could be construed as a potential conflict of interest.

## Publisher's Note

All claims expressed in this article are solely those of the authors and do not necessarily represent those of their affiliated organizations, or those of the publisher, the editors and the reviewers. Any product that may be evaluated in this article, or claim that may be made by its manufacturer, is not guaranteed or endorsed by the publisher.

## Supplementary Material

Supplementary Figure 1 Schematic overview. Schematic overview of experimental series for the imaging of angII-induced AAA/AAD formation in apoE^−/−^ mice. First a basal MRI characterization was conducted before implantation of the osmotic minipumps. PFCs were intravenously injected on day 0, 2, and 4 after minipump implantation. ^1^H/^19^F MRI was conducted on day 2, 4, 7, and 10. Finally, the animals were sacrificed, the aorta excised and subjected to flow cytometry, *ex vivo* ^1^H/^19^F MRI and/or histology. Schematic drawing of the mouse was derived from: https://smart.servier.com/.

Supplementary Movie 1 Blood flow in abdominal aortic dissections and aneurysms. Sagittal ^1^H cine FISP movies to visualize the blood flow in AADs (left) and AAAs (middle). Right: Sagittal ^1^H black blood movie. Note the turbulent flow that is visible in the AAA area of both the bright and black blood movie.

Supplementary Movie 2 Spatial distribution of the ^19^F signal in AAD. 3D reconstruction of a segmented high resolution ^1^H/^19^F MRI scan of an excised, fixed, and embedded aorta with aortic dissection. Gray, external area of the vessel; green, lumen of the vessel; “hot-iron”, ^19^F signal.
